# Bacterial vaginosis: a synthesis of the literature on etiology, prevalence, risk factors, and relationship with chlamydia and gonorrhea infections

**DOI:** 10.1186/s40779-016-0074-5

**Published:** 2016-02-13

**Authors:** Christian T. Bautista, Eyako Wurapa, Warren B. Sateren, Sara Morris, Bruce Hollingsworth, Jose L. Sanchez

**Affiliations:** Division of Health Research, Lancaster University, Lancaster, UK; Walter Reed Army Institute of Research, Maryland, USA; Armed Forces Health Surveillance Center and Cherokee Nation Technology Solutions, Maryland, USA

**Keywords:** Bacterial vaginosis, Chlamydia, Gonorrhea, Military, Epidemiology, STI

## Abstract

Bacterial vaginosis (BV) is a common vaginal disorder in women of reproductive age. Since the initial work of Leopoldo in 1953 and Gardner and Dukes in 1955, researchers have not been able to identify the causative etiologic agent of BV. There is increasing evidence, however, that BV occurs when *Lactobacillus spp.*, the predominant species in healthy vaginal flora, are replaced by anaerobic bacteria, such as *Gardenella vaginalis*, *Mobiluncus curtisii*, *M. mulieris*, other anaerobic bacteria and/or *Mycoplasma hominis*. Worldwide, it estimated that 20–30 % of women of reproductive age attending sexually transmitted infection (STI) clinics suffer from BV, and that its prevalence can be as high as 50–60 % in high-risk populations (e.g., those who practice commercial sex work (CSW). Epidemiological data show that women are more likely to report BV if they: 1) have had a higher number of lifetime sexual partners; 2) are unmarried; 3) have engaged in their first intercourse at a younger age; 4) have engaged in CSW, and 5) practice regular douching. In the past decade, several studies have provided evidence on the contribution of sexual activity to BV. However, it is difficult to state that BV is a STI without being able to identify the etiologic agent. BV has also emerged as a public health problem due to its association with other STIs, including: human immunodeficiency virus (HIV), herpes simplex virus type 2 (HSV-2), *Chlamydia trachomatis* (CT) and *Neisseria gonorrhoeae* (NG). The most recent evidence on the association between BV and CT/NG infection comes from two secondary analyses of cohort data conducted among women attending STI clinics. Based on these studies, women with BV had a 1.8 and 1.9-fold increased risk for NG and CT infection, respectively. Taken together, BV is likely a risk factor or at least an important contributor to subsequent NG or CT infection in high-risk women. Additional research is required to determine whether this association is also present in other low-risk sexually active populations, such as among women in the US military. It is essential to conduct large scale cross-sectional or population-based case-control studies to investigate the role of BV as a risk factor for CT/NG infections. These studies could lead to the development of interventions aimed at reducing the burden associated with bacterial STIs worldwide.

## Background

Over the past two decades, several in vitro and in vivo studies have reported that bacterial vaginosis (BV), a common vaginal condition in women of reproductive age, is a biological risk factor associated with transmission of sexually transmitted infections (STIs), including chlamydia and gonorrhea infection. Although the aetiology of bacterial vaginosis is still unknown, it is believed that occurs when *Lactobacillus spp*., the predominant species in healthy vaginal flora, is replaced by anaerobes, mainly *Gardnerella vaginalis*. The purpose of this review is to provide information that clearly discusses on etiology and epidemiology of BV, and especially its association with transmission of chlamydia and gonorrhea infections. For STI control efforts, the role of BV on these common bacterial infections is an important area for research because there is evidence from randomized trials that screening and treating of asymptomatic women with BV reduce the risk of infection.

## Bacterial vaginosis

### Etiology

In 1892, Doderlein identified, by culturing vaginal secretions of healthy women, *Lactobacillus spp.*, a gram-positive facultative anaerobic bacteria first discovered in sour milk by Scheele in 1780 and subsequently in humans by Folwarczny in 1858 [1]. In 1921, Schroeder confirmed Doderlein’s findings and developed three grades to assess the microbial composition of the vaginal flora. These grades are as follows: 1) healthy vaginal microflora (grade I); 2) *Lactobacillus spp.* partially replaced by other bacteria (grade II), and 3) *Lactobacillus spp.* completely replaced by other bacteria (grade III) [2]. Seven years after Schroeder’s work, Thomas identified Doderlein’s bacteria by microscopy and culture as *Lactobacillus acidophilus* [3]. Based on these findings, Thomas and Schroeder suggested that vaginal discharge is associated with a lactobacilli-deficiency.

Until the mid-1950s, physicians used the term “non-specific vaginitis” for women with lactobacillary grade III vaginal discharge without yeast cells or *Trichomonas vaginalis* [1]. In 1953, Leopold, a US Army captain, was the first to isolate and describe a small, gram-negative, nonmotile, nonencapsuled, rod-shaped bacterium from cervical swabs of women with cervicitis and from men with prostatitis. Although he did not name its species, he suggested that the organism was a member of the genus *Haemophilus* [4]. Two years later, Gardner and Dukes isolated the organism reported by Leopold in women with non-specific bacterial vaginitis, and based on its origin, the organism was named *Haemophilus vaginalis*. They were also the first to describe *H. vaginalis* as the causative agent of non-specific vaginal infections [5]. In 1978, Pheifer confirmed the findings of Leopold, Gardner and Dukes after treating women with non-specific vaginitis with metronidazole, an antimicrobial drug used to treat bacterial infections [6]. Since the first report of Gardner and Dukes in 1955, numerous researchers have identified *H. vaginalis* in women attending venereal disease clinics in the US [7, 8].

Questions have been raised about the identification of *H. vaginalis* as the etiologic agent of non-specific vaginitis since the initial publication by Gardner and Dukes [9]. In the first study by Gardner and Dukes, 2 out of 13 healthy women became infected after being inoculated with a pure culture of *H. vaginalis*. In their second study, 7 out of 29 women inoculated with *H. vaginalis* became infected. In their third study conducted with 15 healthy women who were inoculated intravaginally with *H. vaginalis*, 11 developed the infection. Combined, these experiments showed approximately 35 % of inoculated women became infected with *H. vaginalis*. For Gardner and Dukes, the isolation of *H. vaginalis* in women inoculated with the bacteria was sufficient proof to postulate that *H. vaginalis* was the etiologic agent of non-specific vaginitis [10]. Nevertheless, most researchers rejected this finding of causality because it did not meet Koch’s postulates, proposed in 1890, on the cause-and-effect relationship between bacteria and disease [11].

In the 1960s, there was considerable criticism on whether this small anaerobic bacterium belonged to the genus *Haemophilus*. In 1963, Zinnemann and Turner proposed renaming *H. vaginalis* as *Corynebacterim vaginale* because it has a corynebacterium-like morphology [12]. In 1979, Greenwood and Pickett indicated that *H. vaginalis* had no established genus, and thus, a new genus should be established for this bacterium [13]. Using several biochemical methods, such as DNA-DNA hybridization, biochemical analysis of the cell wall, and electron microscopy, in 1980, Greenwood and Pickett showed that *H. vaginalis* did not belong to the genus *Haemophilus* and renamed it *Gardnerella vaginalis* in honor of Gardner who had first reported the association between non-specific vaginitis and this bacteria [14]. In the same year, the name *G. vaginalis* was also supported by a second taxonomic study conducted by Piot [15]. Since 1983 physicians have used the term “bacterial vaginosis” to differentiate the vaginal discharge syndrome described by Gardner and Dukes from those caused by other microorganisms (e.g., parasites or fungi).

Other microorganisms in the vaginal microflora besides *G. vaginalis* have been discovered in the past two decades. These include *Mobiluncus curtisii*, *M. mulieris*, *Mycoplasma hominis* and anaerobic bacteria, such as *Bacteroides spp.*, *Prevotela spp.*, *Peptostreptococcus spp.*, *Fusobacterium spp.*, and *Porphyromonas spp* [1]. *G. vaginalis* has also been detected in culture samples from nearly all symptomatic women with bacterial vaginosis (BV) and in approximately 50 % of the vaginal microflora of healthy women [16]. Reasons why other species of the genus *Lactobacillus* (e.g., *L. gasseri*, *L. jensenii*, *L. iners*, and *L. crispatus*), that also produce hydrogen peroxide (an antimicrobial product protecting against deleterious microorganisms), lactic acid (which maintains the normal vaginal pH balance between 3.5 and 4.5), and bacteriocins (homegrown antibiotics that inhibit the growth of harmful organisms within the vagina) are superseded by other pathogens (e.g., *G. vaginalis*) are unknown, as is their overall role in the vaginal microflora [17]. According to a conceptual model for BV pathogenesis developed by Schwebke in 2014, *G. vaginalis* is the pathogen responsible for the initiation of BV, with other pathogens acting as secondary “intruders” [18]. It is estimated that *Lactobacillus spp*. comprise 90–95 % of the total bacteria count in the healthy vaginal flora of reproductive age women and maintain balance in the vaginal ecosystem [19].

### Prevalence

For clinicians, BV is a common vaginal condition characterized by at least three of the following four Amsel criteria: 1) thin, gray/white discharge; 2) malodorous “fishy” discharge upon adding 10 % potassium hydroxide; 3) high vaginal pH (>4.5), and 4) identification of vaginal epithelial cells heavily coated with bacteria (i.e., “clue cells”) [20]). For research purposes, BV is commonly diagnosed by a Gram’s stain-based evaluation of vaginal bacterial morphotypes using the Nugent score (≥7 indicates BV) [21]. It is assumed that BV is characterized by the replacement of normal vaginal lactobacilli with that of anaerobic microorganisms (e.g., *G. vaginalis, Prevotella, Peptostreptococcus, and Bacteriodes spp)*. This replacement causes an imbalance in the vaginal microflora, which is the pathophysiologic process responsible for the discharge. It is estimated that 20–30 % of women with vaginal discharge have BV, although the prevalence can be as high as 50–60 % in some high-risk sexual behavior populations [16, 22].

A 2013 systematic review reported that BV prevalence varies between and within countries worldwide [23]. Women from South and East Africa have higher rates of BV (68 % in Mozambique, 51 % in Lesotho, 44 % in Kenya, 37 % in Gambia) compared to women from West Africa (7 % in Burkina Faso) [21]. In Norway (24 %), Turkey (23 %), and Poland (19 %), women have moderately high BV rates. Women from Southeast Asia, Australia, New Zealand, and Indonesia have rates of BV that are typically greater than 30 %. Women in Latin America and the Caribbean have lower rates of BV, except in rural and antenatal populations in Jamaica and Peru (rates of −40 %). In the US, BV is a common condition among women, with prevalence varying by race/ethnicity: African-American (51 %), Hispanic (32 %), and whites (23 %). Similarly, in Canada, aboriginal and indigenous women have high BV rates (33 %). Among female military recruits to the US Marine Corps between 1999 and 2000, the prevalence of BV was 27 % [24]. According to this study, Native American (34 %), African-American (32 %), and Hispanic (30 %) female recruits had the highest BV burden. Data from the US Defense Medical Surveillance System indicates that between 2004 and 2013, 149,666 (15,000 cases per year) BV cases were reported in women in the US military. Of these, 45 % occurred among U.S. Army personnel (unpublished data).

A recent ecological study conducted by Kenyon and Colebunders among males in 11 countries (Central African Republic, Brazil, Cote d’Ivoire, Kenya, Lesotho, Philippines, Singapore, Sri Lanka, Thailand, Zambia, and Tanzania) reported a moderate correlation between the number of partners (as measured by the question, “Do you now have one or more than one spouse/regular partner?”) and BV prevalence (R^2^ = 0.57) [25]. Recent studies conducted among pregnant, HIV-positive and infertile women have also reported high BV prevalences. Among pregnant women in northeastern Nigeria and Ethiopia, the prevalence of BV was 17 and 19 %, respectively [26, 27]; among human immunodeficiency virus (HIV) positive women, a BV prevalence of 48 % has been described in India [28], whereas among infertile women in Qom and Iran the prevalence of BV was found to be 70 % [29]. Information on the burden of BV in Eastern Europe is limited. However, in Bulgaria, by polymerase chain reaction (PCR) and Gram’s staining methods, the BV prevalence was found to be 56 and 57 %, respectively [30]. It has also been reported that the prevalence of BV is high among women with tubal factor infertility in Nigeria [31].

In recent years, BV among women who have sex with women (WSW) has received additional research attention. Between 1995 and 2014, five studies have reported high prevalence estimates (~25 to ~50 %) among WSW [32]. Although there is no specific anatomic or physiologic reason to explain this high prevalence, it has been hypothesized that vaginal fluid exchange may represent an efficient mode of transmission, much as occurs with penile-vaginal sex. Researchers believe that WSW are also a unique high-risk population for the study of BV pathogenesis. Moreover, there is evidence that sexual relationships and behaviors have a strong influence on BV acquisition. These findings would support the hypothesis that BV is sexually transmitted.

### Epidemiology and risk factors

Several studies have identified numerous sexual risk behaviors and other risk factors associated with BV [32–48]. Women are more likely to suffer from BV if they: 1) report a higher number of lifetime sexual partners: 2) are unmarried; 3) were at younger at their first intercourse; 4) self-identify as commercial sex workers (CSWs), and 5) practice regular douching. Other epidemiologic risk factors which have been implicated to a lesser degree include: 1) a high frequency of vaginal intercourse (“state frequency”); 2) a history of pregnancy, and 3) cigarette smoking [35]. In 2014, Jespers et al., reported that recent unprotected sex within 14 to 72 h prior to sampling was a risk factor for BV among CSWs in Kenya, Rwanda, and South Africa; unprotected sex was measured by the presence of prostate-specific antigen in the vaginal fluid [37]. This finding added to the literature the finding that in high-risk populations, male semen and vaginal penetration influence the development of BV. A recent systematic review and meta-analysis identified female genital mutilation or cutting as a risk factor for BV development [38]. In addition, a secondary data analysis of HIV-negative CSWs from South Africa, Uganda, Benin, and India showed that recent vaginal cleansing increases the risk of BV recurrence and that consistent condom use decreases the risk of BV [39]. To date, there is no clear evidence that BV is associated with pelvic inflammatory disease, although several studies have reported a link between BV and cervicitis [40].

A recent large cross-sectional study conducted among 53,652 rural married women in China reported that over 35 days of the menstrual cycle, less than 3 days of menstruation, dysmenorrhea, and usage of an intrauterine device were associated with BV [41]. Other studies have also reported that personal hygiene behaviors, such as vaginal douching, are consistently associated with BV [42]. On the other hand, current use of hormonal contraception, the luteal phase of menstrual cycle, a higher income, and vaginal candidiasis have been reported as protective factors for BV among women attending cervical screenings in south eastern Brazil [43]. Findings from a large cohort study conducted among CSWs in Uganda indicated that hormonal contraception was a protective factor against BV [44]. This finding has been corroborated in a recent systematic review and meta-analysis, where the authors found that hormonal contraception reduces the risk of BV, and thus has a potential implication for prevention [45]. In addition to hormonal contraception, consistent condom use with a primary partner has been found to prevent the recurrence BV among CSWs, highlighting the importance of this barrier method [46]. Although several studies have found numerous protective and risk factors associated with BV in different risk behavior groups, data on the emotional, sexual and social impact of living with BV is sparse in the literature. A recent study conducted by Bilardi et al. [47] reported that women with recurrent BV felt embarrassed, ‘dirty’, ashamed and worried that others may detect their malodor and abnormal discharge. Therefore, BV affected their self-esteem and sex lives by making them avoid having sexual contact with their partners.

There is additional evidence that oral and anal sex may increase the risk of BV. Among adolescent girls who visited sexually transmitted infection (STI) clinics in Baltimore, Maryland, between 1990 and 2002, BV was reported more often among those who reported oral and anal sex [48]. In another study, receptive anal sex before vaginal sex was independently associated with BV among 773 young sexually active women 18–30 years of age [49]. In high-risk groups such as WSW, it was found that the use of vaginal sex toys, oral-anal sex (e.g., anilingus), presence of a BV condition in the female partner and a higher lifetime number of female sex partners increased the risk of BV [50]. A recent study conducted in 355 lesbian and bisexual women using a computer-assisted self-interview approach revealed that presence of *Lactobacillus gasseri* and vaginal lubricant use increased the risk for BV [51]. In a second study among 196 African American WSW, douching within the past 30 days, younger age (18 years or less) at first sexual encounter with a female partner, and two or more male lifetime sexual partners were positively associated with BV [52]. Data from a 24-month WSW cohort study involving visits every 3 months found that receptive oral sex, BV symptom onset, and exposure to a new sexual partner positively influenced BV acquisition. This same study reported that women who co-enrolled with a BV-negative partner reduced their risk of BV acquisition by 74 % [32].

**Fig. 1:**
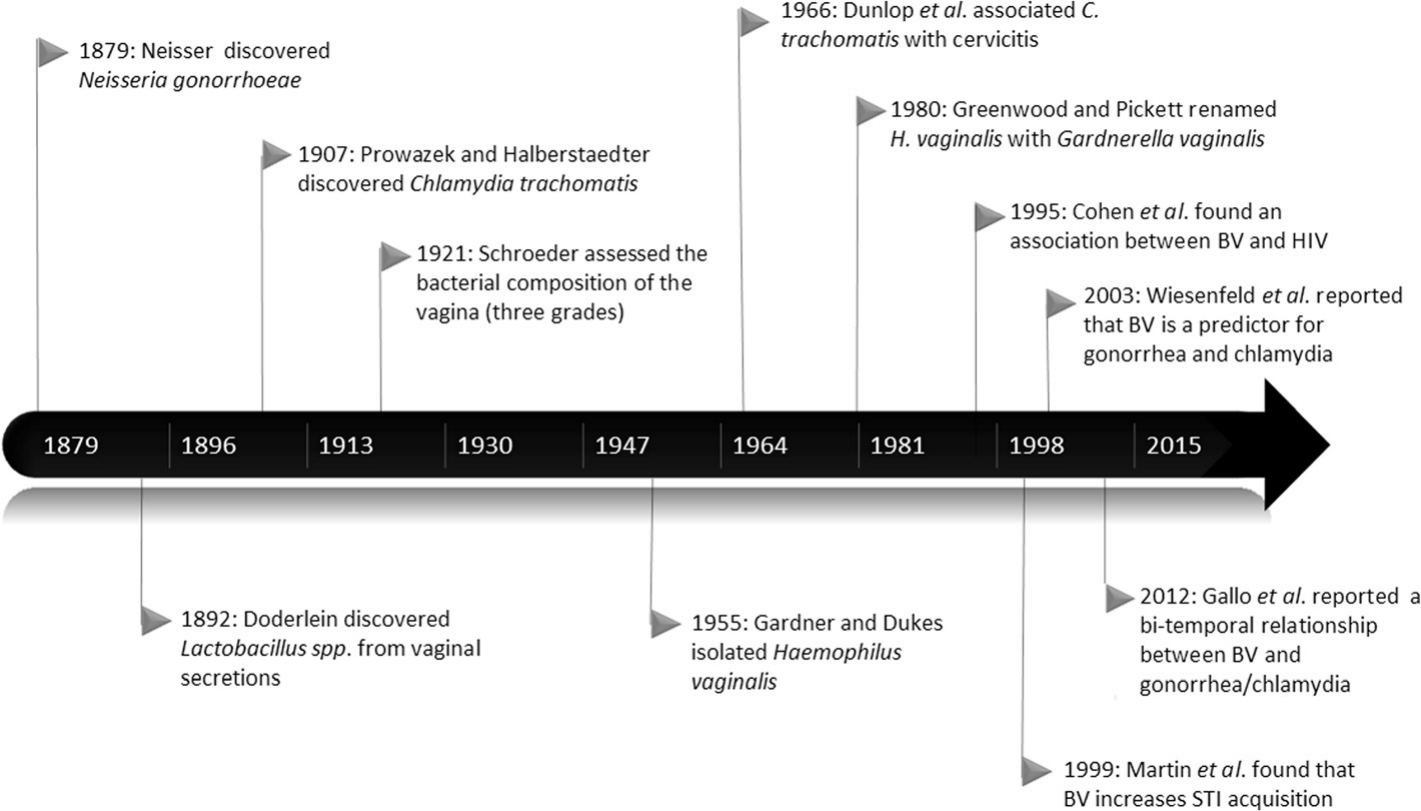
Timeline and milestones related to bacterial vaginosis and associated sexually transmitted pathogens; 1879 to present. Note: BV, bacterial vaginosis; *H. vaginalis, Haemophilus vaginalis*; STI, sexually transmitted infections; HIV, human immunodeficiency virus; *C. trachomatis*, *Chlamydia trachomatis*

Despite 60 years of research since the work of Leopold, Gardner and Dukes in the 1950s, the causative or etiologic agent(s) of BV has not been definitively established. Therefore, it is difficult to state that BV constitutes a STI or condition, although there is growing evidence supporting the hypothesis that BV is sexually transmitted [53]. As an example, in the past decade, numerous studies have reported the contribution of sexual activity to BV, and BV has further emerged as a global issue of concern due to its association with STIs [54]. Fig. 1 shows the timeline of the main milestones of BV and its association with STIs. A 2009 systematic review and meta-analysis on sexual risk factors associated with BV noted that the epidemiological profile of BV is similar to common bacterial STIs, such as chlamydia and gonorrhea. This would support the hypothesis that BV is sexually transmitted and that the number of sexual partners and condom use are risk and protective factors for BV, respectively. For some researchers there is a possibility that sexual activity may be a risk indicator for BV transmission. For instance, Verstraelen stated that BV constitutes a sexually enhanced disease, is not a classic STI and that BV transmission increases with the frequency of sexual intercourse [35]. Verstraelen also agreed with Vallor [55] and Leppaluoto [56], both of whom reported that an increasing frequency of sexual intercourse is associated with an imbalance of the vaginal microflora in favor of BV-related microorganisms. In contrast, Marrazzo suggests that there is evidence to support a role for acquisition of BV, but this process may vary among subsets of women [57]. Elevated cervicovaginal levels of interleukin (IL)-1 beta, IL-6, and IL-8, which are higher in pregnant adolescents with BV, may increase one’s vulnerability to a STI [58].

## Chlamydia and gonorrhea

*Genital Chlamydia trachomatis* (CT) is a common bacterial STI worldwide that is spread through oral, anal, or vaginal sex in both women and men [59]. Based on US National Health and Nutrition Examination Survey (NHANES) data collected from 2007 to 2012, the prevalence of chlamydia has been estimated to be 1.7 % among adults 14–39 years old [60]. Data from NHANES also indicates that African-Americans (5.2 %), people with two or more lifetime sexual partners (3.2 %), divorced/widowed/separated participants (3.0 %), and participants 20–24 years of age (2.9 %) have higher infection rates. In the US, it is estimated that 2.8 million cases of chlamydia are reported each year [61]. According to the Armed Forces Health Surveillance Center (AFHSC), from 2000 to 2012, the human papillomavirus (HPV, 304,021 cases), chlamydia (198,274 cases), and gonorrhea (41,713 cases) were the three most frequently diagnosed STIs among US military personnel on active duty status [62].

One epidemiologic characteristic of a *Chlamydia* infection is that it is usually asymptomatic (~75 % in women, ~50 % in men) [63]. In women, common chlamydia symptoms include abdominal pain, abnormal vaginal discharge, intermenstrual bleeding, painful intercourse, burning while urinating, vaginal bleeding after intercourse, and a yellow discharge with a strong odor [64]. In men, the most common chlamydia symptom is urethritis, followed by a burning sensation during urination, and itching of the skin of the penis. Younger age is the main risk factor associated with chlamydia among women, and 60–70 % of chlamydia infections are reported among adolescents and young adults <25 years of age [59]. Other risk factors associated with infection are: 1) smoking; 2) substance use; 3) preceding HPV infection; 4) number of lifetime sexual partners; 5) sex with new partners; 6) lack of condom use, and 7) having a sex partner with a CT infection, cervicitis, and/or prior history of chlamydia or other STI [65, 66]. In women, chlamydia causes cervicitis, urethritis, and endometritis. Untreated cervicitis can cause pelvic inflammatory disease (PID), ectopic pregnancy, infertility, and chronic pelvic pain. In men, untreated chlamydia may cause tender or swollen testicles and a decline in sperm mobility and concentration, which are both associated with infertility [66].

*Neisseria gonorrhoeae* (NG) is a bacterium that causes gonorrhea, a curable and very contagious infection transmitted through genital and anal sex and less frequently through oral sex [67]. It constitutes the second most common STI worldwide accounting for ~100 million of the estimated ~500 million new cases of curable STIs worldwide annually [68]. In the US, approximately one million cases were reported at the disease’s peak in 1975, with decreasing incidence to 350,000 cases by the year 2000. The principal factor in gonorrhea’s decline was the widespread use of penicillin as a first line antimicrobial in the mid-1970s [69]. In 2013, the national incidence of gonorrhea was 106.1 cases per 100,000 persons. Of the 333,004 cases reported that year, 93 % occurred in persons aged 15–44 years. Gonorrhea is also commonly reported among active duty personnel in the US Armed Forces, with stable incidence rates since 2001 (~200 cases per 100,000 persons-years) [62].

In men, gonorrhea most commonly presents as acute urethritis. Asymptomatic infection rates may be as high as 75 %, in much the same manner as chlamydia infections are for women. In women, the bacteria initially infect the endocervical canal causing gonococcal cervicitis; however, gonorrhea is asymptomatic in up to 70–90 % of women, providing an important reservoir of infection [70]. When symptomatic, common signs and symptoms in women are vaginal discharge (green or yellow color) with an unpleasant odor, together with bleeding during sexual intercourse, painful urination, or itching.

For both men and women, the strongest risk factor associated with a NG infection is young age (15–24 years) [71]. Other risk factors/indicators include: 1) African-American or Hispanic race/ethnicity; 2) illicit drug use; 3) casual sex partners; 4) presence of other STI pathogens (e.g., chlamydia); 5) history of STIs; 6) lack of barrier contraception, and 7) inconsistent condom use [72, 73]. Biological, behavioral, and socio-cultural factors influence the risk of gonorrhea transmission in adolescents and young adult populations [74–76]. First, compared to older women, younger women have larger areas of cervical ectropion and thus are biologically more susceptible to infection. Second, sexually active younger populations engage in more risky sexual behaviors (e.g., multiple sexual partners) compared to the general population, increasing the risk of infection. Finally, at the community level, a gender imbalance represents a factor for transmission; in areas with more women than men, young women are vulnerable to subtle coercion to engage in high-risk sexual behaviors.

In the past decade, a new generation of non-culture tests called nucleic acid amplification tests (NAATs), which are both highly sensitive and specific, have revolutionized diagnosis of gonorrhea and chlamydia infections [77]. Two valuable characteristics of NAAT ‘testing’ are the use of less invasive samples, including self-collected specimens such as urine samples, and the possibility of detecting both gonorrhea and chlamydia using the same specimen [78].

## Bacterial vaginosis and associations with HIV, chlamydia, and gonorrhea

In 1995, Cohen reported a positive relationship between BV and HIV in CSWs from Chiang Mai, Thailand [79]. Since then, this association has been reported in pregnant and postnatal women in Malawi, women in South Africa, and CSWs in Kenyan, among others [80–82]. A systematic review of 23 cohort studies estimated that BV increases the risk of HIV by 60 % [83]. Although causality remains unclear, a high pH in the vaginal environment (>4.5) may allow for the adherence and survival of HIV, increasing the risk of HIV infection. Other studies also indicate that BV is a risk for both ulcerative (e.g., herpes simplex virus type 2 [HSV-2], syphilis [84] and non-ulcerative STIs (e.g., gonorrhea, chlamydia).

The relevant epidemiological studies published on BV, CT, NG and HIV are shown in Table 1. Joesoef in 1996 was the first to report an association of BV with chlamydia (two-fold increase) and gonorrhea (six-fold increase) among pregnant women with BV in Indonesia and suggested BV as a potential marker for these two common bacterial STIs [85]. One year later, Keane conducted a case-control study among 51 couples and observed a strong association between chlamydia and BV (odds ratio = 5.4) [86]. That same year, a large Swedish cross-sectional study involving 1101 women found that high-risk sexual behaviors, such as a high number of lifetime sexual partners, a history of anal sex, having multiple partners in the last month, and a history of sexual abuse and rape were similar among both BV and chlamydia patients [87]. In 1999, Martin found that absence of vaginal lactobacilli increased the risk of gonorrhea (hazard ratio = 1.7), but not chlamydia [88]. Wiesenfeld reported a strong relationship between BV and both CT and NG infections following exposure to male partners with urethritis; this study was conducted among 255 non-pregnant women 15–30 years of age [89]. In this study, women with BV were 4.1 times more likely to test positive for NG and 3.4 times more likely to test positive for CT compared to women without BV. Among 1179 African-American women who were followed for 3 years with visits every 6 and 12 months between 1999 and 2001, Ness et al., found that BV was associated with concurrent NG and CT infections at baseline but not subsequently [90]. However, the authors conducted a trial of NG/CT treatment during follow-up and this might have affected their estimates. According to a secondary data analysis of 535 women at high-risk for STI, Allsoworth observed that BV severity, as measured by a high Nugent score (8–10), was associated with incident STIs (NG, CT and *Trichomonas vaginalis*) and with severe BV cases experiencing a 2-fold increased risk for STIs compared to women with normal vaginal flora [91].

**Table 1:** Summary of studies evaluating the association of bacterial vaginosis with *Chlamydia trachomatis* and *Neisseria gonorrhoeae*

Reference	Location	Study design	Population	Findings
Joesoef, et al. [85]	Indonesia	Cross-sectional	Pregnant women	Women with BV had more than a 2-fold increase in chlamydia and a 6-fold increase in gonorrhea
Keane, et al. [86]	London, UK	Case-control	Women attending genitourinary medicine clinics	Association between chlamydia and BV (odds ratio = 5.4)
Nilsson, et al. [87]	Stockholm, Sweden	Cross-sectional	Women attending family planning and youth clinics	BV is associated with sexual behavior risk factors similar to those associated with *Chlamydia*
Martin, et al. [88]	Mobasa, Kenya	Cohort	Sex workers	Absence of vaginal lactobacilli increased the risk of gonorrhea (hazard ratio = 1.7)
Wiesenfeld, et al. [89]	Pennsylvania, US	Cross-sectional	Non-pregnant women who sought care at STD clinics	Women with BV were more likely to test positive for *N. gonorrhoeae* (odds ratio = − 4.1 or *C. trachomatis* (OR = 3.4)
Ness, et al. [90]	Pennsylvania, Colorado, California, Alabama, South Carolina, US	Cohort	Women visiting planning clinics, university health clinics, gynecology clinics, and STD units	Baseline BV prevalence was associated with gonococcal or chlamydial genital infection (OR = 2.8)
Allsworth, et al. [91]	Rhode Island, US	Cohort	Women attending primary care, gynecology, and family planning clinics	Severity of BV (Nugent score >8) was associated with the incident of a STI (*C. trachomatis, N. gonorrhoeae, Trichomonas vaginalis*, or pelvic inflammatory disease)
Brotman, et al. [92]	Alabama, US	Cohort	Non-pregnant women visiting clinics for routine care	BV at the prior visit increased the risk of a subsequent *C. trachomatis* (hazard ratio = 1.9) and *N. gonorrhoeae* (hazard ratio = 1.8) infection
Gallo, et al. [93]	Alabama, US	Cohort	Women attending public STD clinics	BV increased the risk of gonorrhea/chlamydia (pairwise odds ratio = 1.6) and gonorrhea/chlamydia also increased the risk of BV (pairwise odds ratio = 2.4)

Findings from two cohort studies associating BV with a CT or NG infection were published in 2010 and 2012 [92, 93]. Both studies were conducted among non-pregnant women. According to Brotman’s study, which was a secondary analysis of 3620 non-pregnant women 15–44 years of age enrolled in the Longitudinal Study of Vaginal Flora in Birmingham, Alabama from 1999 to 2002, women with BV (measured as a Nugent score of 7–10) had a 1.8- and 1.9-fold increased risk for gonorrhea and chlamydia, respectively. The second cohort study conducted by Gallo re-analyzed data from a large condom intervention study among 1159 women who attended public STI clinics between 1995 and 1998 in Birmingham and Huntsville, Alabama, and used generalized estimating equations methods to account for multiple individual visits (6 monthly follow-up visits). The authors reported that women with an incident BV episode at a prior visit were at a 1.6 times the risk of having chlamydia or gonorrhea at a subsequent follow-up visit. In addition, Gallo found a relationship between these two bacterial STIs and BV; namely, chlamydia or gonorrhea increased by 2.4 times the risk of having BV at a subsequent visit. This represents the first report of a temporal relationship working in both directions between BV and chlamydia/gonorrhea. Recently, two studies have reported a significant association between BV and these STIs among women in Durban, South Africa and among female sex workers in Uganda [58, 94].

Considered together, multiple studies report BV as a risk factor or at least an important contributor in subsequent gonorrhea or chlamydia infection. It is important to note that the observation that BV increases the risk of chlamydia or gonorrhea was mainly seen in high-risk women (e.g., CSWs, women attending STI clinics, and/or women at risk for unplanned pregnancies). Additionally, the two recent cohort publications are based on secondary data analyses of data collected in the late 1990s to early 2000s, and their findings must be interpreted with caution considering the changes in the epidemiology of chlamydia in the US over the past decade. We believe that additional research with increased sample sizes and using modern epidemiological and statistical methods in other young sexually active women affected by these bacterial STIs are warranted. For instance, women serving in the US Armed Forces can provide additional data-based evidence about the role of BV as a risk factor or indicator for CT and NG infection. In addition, molecular studies to determine risk factors and adverse outcomes associated with the subtypes of BV in different risk behaviors groups are also required. Data from these studies may provide additional evidence as to the epidemiology of this leading disorder among women of reproductive age.

## Prevention implications

Although a definitive causative agent for BV has not yet been shown, there is evidence, albeit among higher risk groups, that the presence of BV is positively associated with both CT and NG occurrence. Existing findings to date suggest that patients, clinicians and public health personnel should be made aware of the positive association of BV with CT and NG, and given the clustering of risk behaviors behind both, should pay additional attention to education on sexual hygiene measures (i.e., douching as a risk factor for BV) and sexual risk behaviors for both BV and CT/NG (i.e., younger age at sexual intercourse, higher number of sexual partners, sex with partner with STI history, and most importantly the lack of barrier contraception). A clinician diagnosis of BV can be a risk alert for eventual patient acquisition of STIs and counseling is suggested, with elevated concern for women presenting with repeat BV infections. Despite the potential for prevention measures associated with the identification of BV as a risk indicator for common STIs, there is still the need for further research among young women not in high risk sexual behavior groups. Notwithstanding the need for additional research information, data to date suggest a role for BV diagnosis, treatment and prevention in STI prevention.

## Conclusion

Since the discovery by Leopold in 1953 of the microorganism associated with non-specific vaginitis, now named *Gardnerella vaginalis*, there has been increasing evidence that the replacement of vaginal lactobacilli with this microorganism is associated with BV and that it is the most common cause of abnormal vaginal discharge among women of reproductive age. Although the epidemiological profile of BV seems to be similar to chlamydia and gonorrhea, this cannot be interpreted as evidence that BV is an STI. In the past decade, numerous studies have reported an association between BV and STI pathogens, such as HIV, HSV-2, CT, and NG. The biological mechanisms underlying such an association are still unknown; however, there is growing evidence supporting the hypothesis that BV increases the risk of acquiring STIs. Studies relating BV to chlamydia or gonorrhea were conducted mainly among non-representative high-risk populations; thus, additional research is required to determine whether these associations are also present in other young sexually active populations (e.g., among women in the US military). The effect of BV on chlamydia or gonorrhea is an interesting area for future research because there is evidence from two randomized trials that screening and treating asymptomatic women with BV reduces the risk of acquiring a CT or NG infection. Twice-weekly prophylactic use of intravaginal metronidazole gel significantly reduced the incidence of CT infections among women attending a STI clinic in Alabama [95], and the incidence of CT and NG infections was lower among US and African women who used intravaginal metronidazole/miconzaole compared to the placebo group [96]. Therefore, to determine the role of BV as a risk factor for CT/NG infections, it is essential to conduct large studies to provide solid epidemiological evidence in favor of such associations.

### Ethical approvals

This study protocol was reviewed and approved by institutional review boards at Lancaster University and Walter Reed Army Institute of Research.

## Abbreviations

AFHSC: Armed Forces Health Surveillance Center
BV: bacterial vaginosis
CSW: commercial sex worker
CT: chlamydia trachomatis
DNA: deoxyribonucleic acid
HIV: human immunodeficiency virus
HSV-2: herpes simplex virus type 2
NAAT: nucleic acid amplification test
NG: neisseria gonorrhoeae
NHANES: National Health and Nutrition Examination Survey
PCR: polymerase chain reaction
PID: pelvic inflammatory disease
STI: sexually transmitted infection
US: United States
WSW: women who have sex with women
